# Heat exposure and bicycling trigger recurrent aseptic meningitis: a case report

**DOI:** 10.1186/s12883-014-0230-z

**Published:** 2014-12-31

**Authors:** Olaf Stuve, Ellen Marder, Annette Okai, Mark Stettner, Bernd C Kieseier

**Affiliations:** Department of Neurology and Neurotherapeutics, University of Texas Southwestern Medical Center at Dallas, Dallas, TX USA; Neurology Section, VA North Texas Health Care System, Medical Service, 4500 South Lancaster Rd, Dallas, TX 75216 USA; Department of Neurology, Klinikum rechts der Isar, Technische Universität München, München, Germany; Department of Neurology, Heinrich Heine University Düsseldorf, Düsseldorf, Germany; Multiple Sclerosis Treatment Center of Dallas, Dallas, TX USA

## Abstract

**Background:**

Aseptic meningitis associated with herpes simplex virus type 2 often has a relapsing-remitting clinical phenotype. Factors that lead to disease activation and reactivation are currently incompletely understood.

**Case presentation:**

We describe the case of a 49-year-old Caucasian man who developed recurrent episodes of herpes simplex virus type 2-associated aseptic meningitis in the setting of heat exposure and bicycling. This case is compelling in that substantial data were available to the examining physicians on the amount of physical exercise and heat exposure. Strenuous physical activities or heat exposure in isolation did not cause re-occurrence of clinical signs and symptoms.

**Conclusions:**

This case illustrates that the dual activation of mechanical and temperature receptors in dorsal root ganglia may lead to the recurrent reactivation and afferent dissemination of latent herpes simplex virus type 2 in some patients.

## Background

Herpes simplex viruses (HSV) are neurotropic viruses that are frequent human pathogens [1]. HSV are DNA viruses that are closely related and display approximately 70% genomic homology. HSV2 is primarily associated with genital herpes, but can also be the cause of recurrent herpes labialis. In addition, HSV2 is the trigger for recurrent aseptic meningitis, which is associated with a lymphocytic pleocytosis and occasionally with large endothelial cells termed Mollaret cells. The first onset of disease can occur many years or decades after the initial exposure and viral latency, and relapses can occur in intervals of weeks, months, or years. Anti-viral therapies do not affect the viral reservoir in dorsal root ganglia.

The seroprevalence for HSV2 is around 20% in the United States [2]. Mechanisms that lead to disease activation are currently incompletely understood. This case report illustrates a case of HSV2 activation and reactivation in the setting of physical exercise that was associated with heat exposure. The combination of mechanical and thermal triggers may have led to activation of latent HSV2 in this patient.

### Case Presentation

We report the case of a 49-year-old Caucasian man who developed an acute severe headache centered in the vertex of his skull in April 2008. At the time, he also felt cognitively impaired. The patient reported a fever of up to 39.7°C (103.5°F), and low back pain and hip pain upon hip extension and flexion. Upon assessment by a neurologist, the patient revealed that he had been bicycling for approximately eight hours and 153 kilometer (95 miles) in heat that exceeded 35°C (95°F) approximately 12 hours prior to the onset of his symptoms. His bicycle computer was interrogated to verify the environmental heat exposure. On physical examination, the patient was found to have mild neck stiffness, as well as a positive Kernig sign [3] and Brudzinski sign [4]. The rest of his general and neurological examination was intact. There were no herpetic skin lesions. A magnetic resonance imaging (MRI) study of the brain was obtained and found to be intact. Analyses of cerebrospinal fluid (CSF) showed 128 white blood cells (WBC) per microliter (μl). A polymerase chain reaction (PCR) for herpes simplex virus type 2 (HSV2) was positive. CSF protein, glucose, red blood cell count, oligoclonal bands, IgG synthesis, IgG index, *Treponema pallidum* agglutination test, *Borellia burgdorferi* IgM and IgG, and PCR for HSV1, human herpesvirus 6, cytomegalie virus, Epstein Barr virus, and Varizella-Zoster virus were all normal or negative. No Mollaret cells within the CSF were reported [5]. The patient had no history of labial or genital herpes. His symptoms subsided within 5 days.

The patient had no history of labial or genital herpes. He was started on ganciclovir 500 mg po BID for treatment and subsequent prophylaxis of aseptic meningitis. His symptoms subsided within 5 days. He discontinued ganciclovir after two years.

Five years later, the patient developed neurological signs and symptoms similar to the ones described above. This time, however, the patient also experienced a severe pain in his mid and lower back. The patient reported that he had just completed a three day bicycle tour, during which he had completed approximately 100 kilometer (62 miles) per day, and had been exposed to heat of 39°C (102.2°F) for more than three hours each day. The heat exposure was again documented by a bicycle computer and the local weather report. A CSF examination showed 298 WBC/μl, the protein was 69 milligram per deciliter, and a PCR for HSV2 was again positive. All other diagnostic tests were within normal limits. The patient was treated with acyclovir 800 mg po q4hours for 7 days. His symptoms substantially improved after ten days. The patient decided against prophylactic pharmacotherapy for lack of evidence [6].

This case is compelling in that substantial data were available to the examining physicians on the amount of physical exercise and heat exposure. It is worth noting that prior to the initial episode and in between the two episodes of aseptic meningitis the patient participated in bicycle races that exceeded the distances that triggered the events described above. However, there was no heat exposure above 30°C (86°F) associated with those bicycle rides. Furthermore, the patient also took part in other strenuous physical activities, including numerous marathons during which he was exposed to heat greater than 30°C (86°F), but suffered no ill effects.

HSV2 is an ubiquitous neurotropic and neuroinvasive virus that becomes latent within dorsal root ganglia to evade immune surveillance [1]. Herpes virus dissemination has been observed with immunoglobulin hypoglobulinemia and dysglobulinemia [7,8], something that was not tested in our patient. The events that specifically trigger a disease recurrence of HSV2 are incompletely understood. The nerve endings of dorsal root ganglion neurons have sensory receptors that are activated by chemical, mechanical, and thermal stimuli [9]. We propose that the dual activation of mechanical and temperature receptors in dorsal root ganglia led to the recurrent reactivation and afferent dissemination of latent HSV2 in this patient. Eventually, there may be exaggerated adaptive and innate immune responses against HSV2 antigenic determinants. Franzen-Rohl and coworkers recently demonstrated that patients with a history of recurrent HSV2-assoicated meningitis displayed elevated T cell proliferation and T helper cell (Th)1 and Th2 cytokine expression when challenged with HSV antigens compared to patients with recurrent HSV2-associated genital infections [10]. There was also an increased natural killer (NK) cell response, an increased expression of toll-like receptor (TLR)3 and −9 by dendritic cells, and an increased TLR-induced alpha interferon responses [10].

## Conclusion

This is the first case to suggest that the combination of heat exposure and bicycling may trigger recurrent episodes of aseptic meningitis insusceptible individuals. The patient was advised to restrict his bicycling activities to days with moderate temperatures.

## Consent

Written informed consent was obtained from the patient for publication of this Case report and any accompanying images. A copy of the written consent is available for review by the Editor of this journal.

## Abbreviations

CSF: Cerebrospinal fluid
HSV: Herpes simplex virus
MRI: Magnetic resonance imaging
NK: Natural killer
PCR: Polymerase chain reaction
Th: T helper cell
TLR: Toll-like receptor
WBC: White blood cells
